# Multiscale patterns and drivers of arbuscular mycorrhizal fungal communities in the roots and root‐associated soil of a wild perennial herb

**DOI:** 10.1111/nph.15088

**Published:** 2018-03-24

**Authors:** Pil U. Rasmussen, Luisa W. Hugerth, F. Guillaume Blanchet, Anders F. Andersson, Björn D. Lindahl, Ayco J. M. Tack

**Affiliations:** ^1^ Department of Ecology Environment and Plant Sciences Stockholm University SE‐106 91 Stockholm Sweden; ^2^ School of Biotechnology Science for Life Laboratory KTH Royal Institute of Technology PO Box 1031 SE‐171 21 Solna Sweden; ^3^ Centre for Translational Microbiome Research Department of Molecular, Tumor and Cell Biology Science for Life Laboratory Karolinska Institutet 171 65 Solna Sweden; ^4^ Département de Biologie Faculté des Sciences Université de Sherbrooke 2500 Boulevard Université Sherbrooke QC J1K 2R1 Canada; ^5^ Department of Soil and Environment Swedish University of Agricultural Sciences Box 7014 SE‐750 07 Uppsala Sweden

**Keywords:** arbuscular mycorrhizal (AM) fungi, community composition, fungal community, Moran's eigenvector maps, plant community, soil microbial community, spatial structure

## Abstract

- Arbuscular mycorrhizal (AM) fungi form diverse communities and are known to influence above‐ground community dynamics and biodiversity. However, the multiscale patterns and drivers of AM fungal composition and diversity are still poorly understood.
- We sequenced DNA markers from roots and root‐associated soil from *Plantago lanceolata* plants collected across multiple spatial scales to allow comparison of AM fungal communities among neighbouring plants, plant subpopulations, nearby plant populations, and regions. We also measured soil nutrients, temperature, humidity, and community composition of neighbouring plants and nonAM root‐associated fungi.
- AM fungal communities were already highly dissimilar among neighbouring plants (*c*. 30 cm apart), albeit with a high variation in the degree of similarity at this small spatial scale. AM fungal communities were increasingly, and more consistently, dissimilar at larger spatial scales. Spatial structure and environmental drivers explained a similar percentage of the variation, from 7% to 25%. A large fraction of the variation remained unexplained, which may be a result of unmeasured environmental variables, species interactions and stochastic processes.
- We conclude that AM fungal communities are highly variable among nearby plants. AM fungi may therefore play a major role in maintaining small‐scale variation in community dynamics and biodiversity.

## Abstract 2

See also the Commentary on this article by https://doi.org/10.1111/nph.15212.

## Introduction

Questions of how organisms are spatially distributed and what drives spatial patterns are central to community ecology (Nemergut *et al*., 2013). While most focus in the past has been on describing patterns above ground, we are now seeing a rise in studies investigating the spatial distribution of soil biota and the mechanisms driving their distribution (Nemergut *et al*., 2013; Tedersoo *et al*., 2014; Davison *et al*., 2015). How organisms are structured spatially will depend on their dispersal ability, interspecies interactions and the abiotic and biotic environments, and each of these potential drivers may regulate community composition at different spatial scales (Levin, 1992). While much is known about the spatial structure and drivers of above‐ground plant‐based communities, we still lack a comprehensive understanding of the multiscale patterns and drivers of plant‐associated communities below ground.

Arbuscular mycorrhizal (AM) fungi are root symbionts forming associations with the majority of plants (Smith & Read, 2008). These ubiquitous organisms are important as they facilitate plant uptake of growth‐limiting nutrients, in particular phosphorus, which can affect plant performance (Smith & Smith, 2011). In addition, increases in AM fungal diversity have been linked to increasing plant diversity, growth (van der Heijden *et al*., 1998; Hartnett & Wilson, 1999), and resistance to pathogens and drought (Newsham *et al*., 1995; Augé, 2001). There is also increasing evidence that the impact of AM fungi on plant defence and fitness depends on the composition of the AM fungal communities (Klironomos, 2000; Bever, 2002; Klironomos *et al*., 2004).

A global assessment of AM fungal communities showed low endemism, with the majority of AM taxa found on several continents (Davison *et al*., 2015; but see Bruns & Taylor, 2016; and Bruns *et al*., 2018). While this indicates that dispersal at a global scale does occur, it is generally thought that AM fungal communities are structured by local environmental filtering. However the key drivers may vary across spatial scales. AM fungi are dispersed by hyphal spread over smaller areas and by passive dispersal of spores by wind and animals, in some cases up to 2 km (Warner *et al*., 1987; Mangan & Adler, 2000). Previous work has found large variation in fungal community structure at the submetre scale (Anderson *et al*., 1983; Wolfe *et al*., 2007; Mummey & Rillig, 2008), while clustering and spatial autocorrelation were typically found up to the 1 m scale (Anderson *et al*., 1983). However, many of the studies on the spatial structure of AM fungi have focused on either small (1–2 m; Wolfe *et al*., 2007; Mummey & Rillig, 2008) or global scales (Davison *et al*., 2015), with few studies focusing at the mesoscale (cm to km). In addition, many studies have investigated the AM fungi in roots and soil associated with a mix of plant species (Anderson *et al*., 1983; Whitcomb & Stutz, 2007; Mummey & Rillig, 2008), and variation in AM fungal communities across spatial scales may thus be attributable to variation in plant species identity, rather than the impact of spatial scale *per se*. To the best of our knowledge, only a handful of studies have investigated the spatial structure of AM fungi associated with specific host plant species (Table 1). One study found that spatial autocorrelation accounted for 24% of the variation in AM fungal community composition associated with *Zea mays* in agricultural fields in Zimbabwe (Table 1; Lekberg *et al*., 2007). In another study, which investigated multiscale patterns of AM fungal communities, Chaudhary *et al*. (2014) found that AM fungi associated with two *Artemisia* species were similar at the smallest spatial scale (1 m apart) but varied significantly at larger scales, with the strongest differences between regions (*c*. 50–100 km apart; Table 1). To bridge the gap between small‐ and large‐scale studies, and disentangle the effect of spatial scale from that of plant species identity, in this study we sampled AM fungal communities associated with a single plant species across multiple spatial scales, ranging from tens of centimetres to tens of kilometres.

**Table 1:** Overview of studies on the spatial structure of arbuscular mycorrhizal (AM) fungi associated with a specific host plant

Study	Plant species	Vegetation type	AM fungi measured	Spatial scale measured		Spatial structure	Abiotic factors
				Sample area	Distance between samples		
Chaudhary *et al*. (2014)	*Artemisia filifolia* and *Artemisia* *tridentata*	Semiarid shrubland (UT, USA)	Spore identification	Samples taken in nested design within: (i) four regions (5000 ha); (ii) three sites within regions (1 ha); (iii) two microsites within sites (1 m^2^)	(i) Regions were *c*. 50–100 km apart; (ii) sites were a few km apart; (iii) microsites were up to 1 m apart Nine random samples were taken within each 1 m microsite	Positive spatial autocorrelation. Within‐group heterogeneity of AM fungal community composition higher for regions	P, mean annual temperature and precipitation, elevation and latitude influenced AM fungal community composition. pH, P, electric conductivity, and mean annual temperature influenced AM fungal spore richness. P influenced AM fungal spore evenness
Friese & Koske (1991)	*Ammophila breviligulata*	Sand dune (RI, USA)	Spore identification	Samples taken within: (i) three plots of 40 × 40 × 40 cm	(i) Distance between plots not listed. Sixteen samples were taken from each plot. Samples were 10 × 10 cm taken in 5 cm vertical increments	No spatial autocorrelation, but spores tended to aggregate	No correlation to physical factors found
Hazard *et al*. (2013)	*Trifolium repens* and *Lolium perenne*	Pastures, arable fields, peatlands, forests (Republic of Ireland)	Molecular methods	Samples taken within: (i) 40 sites of 30 m × 30 m	(i) Sites were 7–392 km apart. Five randomly collected root samples (pool of five plants) were taken within each site	No spatial autocorrelation	pH, rainfall and soil type influenced AM fungal community composition
Horn *et al*. (2017)	*Festuca brevipila*	Nature protection area of steppes and coastal habitats (Germany)	Molecular methods	Samples taken in nested design within: (i) three macroplots (15 × 15 m) (ii) four plots within macroplots (3 × 3 m)	(i) Macroplots were 20–500 m apart; (ii) plots were 9–15 m apart. Five samples were taken within plots 30 cm–3 m apart	The spatial structure explained up to 31% of the variation in the AM fungal community, with further variation explained by spatial‐phylogenetic effects	Environmental factors (pH, water content, C, N, C : N ratio, P, dehydrogenase activity) explained up to 10% of the variation in AM fungal community composition
Lekberg *et al*. (2007)	Maize (*Zea mays*)	Semi‐arid agricultural field (Zimbabwe)	Molecular methods	Samples taken within: (i) 10 fields (0.5 ha each)	(i) Fields were 25 km apart Twenty randomly collected root samples were taken within each field	Positive spatial autocorrelation; 23.5% of variation in community composition explained by spatial autocorrelation	Soil variables (texture, moisture, organic C, pH, P, N) explained 38.6% of the variation in AM fungal community composition.
Sylvia (1986)	*Uniola paniculata*	Coastal foredunes (FL, USA)	Spore identification	Samples taken in nested design within: (i) two sites (size not listed) (ii) three plots within sites (1 × 2 m)	(i) Sites were *c*. 100 km apart; (ii) plots were 25–100 m apart. Thirty samples were taken along six diagonal transects within each plot. Each transect was 33 cm apart and each sample was 10 cm apart	AM fungal species differed between sites and plots, but no results are reported for within‐plot differences.	–

Arbuscular mycorrhizal fungal species differ in their responses to variation in soil properties, such as moisture, temperature and nutrient availability (Chaudhary *et al*., 2008), and this may in turn explain spatial variation in diversity and community composition (Wolfe *et al*., 2007; Hawkes *et al*., 2011; Kivlin *et al*., 2011). Lekberg *et al*. (2007) found that 38% of variation in AM fungal community composition in *Zea mays* was explained by the abiotic soil environment (Table 1). However, the biotic environment, including neighbouring plants and nonAM fungi in roots, may also be linked to the diversity and composition of AM fungal communities. While AM fungi are considered to colonize a wide range of host plants (Allen *et al*., 1995; Rosendahl, 2008; Helgason & Fitter, 2009), AM fungi in natural communities associate to a different degree with different host plant species, with some evidence of specialization (Helgason *et al*., 1998; Klironomos, 2000; Bever, 2002). Rodríguez‐Echeverría *et al*. (2017) found that vegetation type influenced AM fungal community composition in tropical African environments, and studies linking AM fungal and plant richness have found positive (Vogelsang *et al*., 2006; Hiiesalu *et al*., 2012), negative (Antoninka *et al*., 2011) or no relationships (Öpik *et al*., 2008, 2010; Lekberg *et al*., 2013). Another structuring factor may be the presence of root‐associated nonAM fungi. For example, Larimer *et al*. (2012) studied the interactions between AM fungi and endophytes in the grass *Elymus hystrix* and found that endophytes influenced AM fungal colonization while AM fungi affected fungal endophyte fitness. Other studies have shown similar reciprocal interactions between AM fungi and fungal pathogens (Newsham *et al*., 1995; Borowicz, 2001). It may thus be important to determine the relationship between different fungal groups in order to understand the community composition of AM fungi.

The community composition of AM fungi may differ between roots and the root‐associated soil. In general, communities of AM fungi found in soil have been considered to represent a common species pool for the entire plant community, with only a subset able to colonize the roots of a given plant species (Johnson *et al*., 2003; Davison *et al*., 2011). However, species diversity in the roots may also be higher than in soil if some species invest more in intraradical colonization with limited extraradical growth (Jakobsen *et al*., 1992; Munkvold *et al*., 2004), thereby limiting species detection in soil. Studies have found both higher (Hempel *et al*., 2007), similar (Busby *et al*., 2013), or lower (Saks *et al*., 2014; Varela‐Cervero *et al*., 2015) richness in soil as compared with roots.

We explored the multiscale patterns and drivers of root and root‐associated soil AM fungal communities associated with *Plantago lanceolata* in naturally fragmented plant populations across the Åland Islands (Finland). We assessed the spatial distribution of root colonization intensity, diversity indices and AM fungal community composition, in both roots and root‐associated soil, on scales ranging from neighbouring plants to regions. We further determined how much of the variation in AM fungal root colonization intensity, diversity indices and community composition was determined by spatial structure and the abiotic and biotic environments. More specifically we asked:

- How does root colonization, diversity indices and composition change with distance across spatial scales? Is most variation found among neighbouring plants (*c*. 30 cm), among plant subpopulations (*c*. 10 m), among plant populations (*c*. 5 km), or among regions (*c*. 30 km)?
- How do the abiotic (soil nutrients and climate) and biotic (i.e. neighbouring plants and nonAM root‐associated fungi) environments correlate with AM fungal root colonization, diversity indices and community composition, and how does this variation interact with spatial structure?
- Do root and soil communities differ?

## Materials and Methods

### Study system and hierarchical sampling design

**Figure 1:**
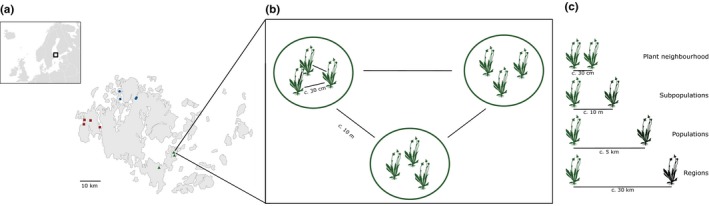
Location of sampled *Plantago lanceolata* individuals in the Åland Islands in the Baltic Sea, southwestern Finland. The top‐left inset shows the location of the Åland Islands within northern Europe. (a) Within the Åland Islands, three to four plant populations were sampled in three regions (with colours and symbols representing plant populations in different regions). (b) The inset illustrates that, within each plant population, we sampled three nearby plant individuals (separated by *c*. 30 cm) in each of three subpopulations within the plant population. (c) Schematic representation illustrating that individual plants at the neighbourhood scale were separated by *c*. 30 cm, plant subpopulations were separated by *c*. 10 m, plant populations within a region were separated by *c*. 5 km, and regions were separated by *c*. 30 km.

To investigate how communities of AM fungi vary across spatial scales, we focused on the perennial herb *Plantago lanceolata* L. in the Åland Islands, southwestern Finland (Fig. 1). This monoecious, rosette‐forming perennial herb has a cosmopolitan distribution and reproduces by outcrossing (Ross, 1973) and frequent clonal propagation through side rosettes (Mook *et al*., 1992). The plant has limited gene flow between populations (Bos *et al*., 1986), and seeds generally disperse within a metre from the mother plant (Bos, 1992). In the Åland Islands, *P. lanceolata* is typically found in small dry meadows (most of them < 1 ha) located within an agricultural landscape (Ojanen *et al*., 2013).

Root and soil samples of individual plants were collected in a nested design at multiple spatial scales (Fig. 1): the regional scale (three regions, *c*. 30 km apart), the population scale (three to four populations within each region, *c*. 5 km apart), the subpopulation scale (distinct sections within plant populations, *c*. 10 m apart), and the neighbourhood scale (individual plants within each subpopulation, *c*. 30 cm apart). In each subpopulation, three *P. lanceolata* plants and the surrounding soil (*c*. 200–500 ml; 25 mm radius around the plant; *c*. 100–150 mm deep) were collected in order to determine root colonization and community composition of root‐associated fungi within both roots and soil. Samples were collected from 7 to 10 July 2015. In total, we sampled 108 plants from 36 subpopulations, 11 populations, and three regions.

Roots were carefully separated from the soil, washed, cut into 2 cm pieces, and mixed thoroughly. Half of the roots from each sample were frozen at −20°C and subsequently freeze‐dried for later sequencing of DNA markers targeting both AM fungi and nonAM root‐associated fungi. The other half of the roots were stored in ethanol for later staining to determine colonization percentage. Roots were stained using trypan blue (Phillips & Hayman, 1970; Koske & Gemma, 1989) and scored using the gridline intersect method (McGonigle *et al*., 1990). The soil was thoroughly mixed and a representative subsample was frozen at −20°C and later freeze‐dried before determination of AM fungal community composition. To determine the community composition of root‐associated fungi in roots and the surrounding soil, DNA was extracted from 10–25 mg freeze‐dried root material (in a few samples below 10 mg) or 250–280 mg freeze‐dried soil using NucleoSpin Plant and Soil kits (Macherey‐Nagel, Düren, Germany).

### Environmental drivers of AM fungal communities

In order to determine the effect of the environment on AM fungi, several abiotic and biotic measurements were conducted. A soil sample (300 ml) from each subpopulation was assessed for pH, plant available P, NH_4_, NO_3_, and total N (by Eurofins, Sweden). To determine the impact of climate, data loggers were placed in a majority of the subpopulations, both above ground (at a height of *c*. 5 cm) and below ground (*c*. 2 cm below the litter layer). Temperature and relative humidity were recorded above ground using Lascar loggers (EL‐USB‐2; Lascar Electronics, Wiltshire, UK), while below‐ground temperature was recorded using iButtons (DS1922L; Maxim Integrated, San Jose, CA, USA). The data loggers were set to record every 2 h for 1 yr, starting in June 2014. Mean below‐ground and above‐ground temperatures and relative humidities were calculated separately for the growing season (April–October) and nongrowing season (November–March). Owing to problems with recovering loggers, we were only able to retrieve data from half of the above‐ground loggers and three‐quarters of the below‐ground loggers. Soil moisture was measured (HH2, SM300; Delta‐T, Cambridge, UK) at three locations around each individual plant in June 2015 and the average value for each plant was calculated.

To investigate the effect of the surrounding vegetation on the community composition of AM fungi, we recorded plant species and their abundance in a 35 × 35 cm square around each sampled plant, and calculated plant species richness, Shannon's diversity index (Shannon, 1948) and Pielou's evenness index (Pielou, 1966). Plant species richness at each sample location was 5.3 ± 2.1 (mean ± SD), with the most common neighbouring plant species being *Achillea millefolium*,* Agrostis capillaris* and *Galium verum*. To investigate the link between AM and nonAM root‐associated fungi, a subset of root samples (*n* = 82) from eight different populations (Supporting Information Fig. S1) were selected.

### Molecular methods

To assess the AM fungal community composition within roots and root‐associated soil, we used the primers NS31 and AML2 targeting a *c*. 560 bp central fragment of the small subunit rRNA gene in the Glomeromycota (Simon *et al*., 1992; Lee *et al*., 2008). These primers have often been used to assess the community composition of AM fungi (Öpik *et al*., 2010; Davison *et al*., 2015). Samples were sequenced at SciLifeLab/NGI (Solna, Sweden) on the MiSeq platform (Illumina Inc., San Diego, CA, USA), and sequences were clustered based on 99% similarity, a cutoff previously used in studies of AM fungal biogeographical patterns (Kivlin *et al*., 2011; but see Bruns & Taylor, 2016; and Bruns *et al*., 2018).To assess nonAM root‐associated fungi we used primers targeting the internal transcribed spacer (ITS) region using the primers fITS7 (Ihrmark *et al*., 2012) and ITS4 (White *et al*., 1990), which target a 250–450 bp fragment encompassing the entire ITS2 with flanking sequences in the 5.8 and large subunit genes. We followed the protocol of Clemmensen *et al*. (2016) and samples were sequenced at SciLifeLab/NGI (Uppsala, Sweden) on a PacBio RS II system (Pacific Biosciences, Menlo Park, CA, USA). Obtained sequences were analysed in the bioinformatics pipeline Scata (http://scata.mykopat.slu.se; Ihrmark *et al*., 2012), whereby they were clustered into operational taxonomic units (OTUs) using single linkage clustering with 98.5% sequence similarity. This resulted in a total of 104 149 sequences, clustering into 1247 OTUs. Sequences were obtained from 77 samples with an average of 1370 reads per sample. For further analyses we used a set of the 179 most common OTUs, making up 90% of the total number of reads (after removing plant and AM fungal OTUs). These OTUs were tentatively identified to the species level by comparing representative sequences with species hypotheses in the UNITE database (Kõljalg *et al*., 2005; Abarenkov *et al*., 2010).

For full details on the molecular methods and bioinformatics, see Methods S1. Sequencing data for each sample in this study have been deposited at NCBI under accession numbers SRP132598 and SRP132591 for root and soil samples, respectively.

### Statistical methods

All analyses were conducted in R v.3.4.2 (R Core Team, 2017). In all multivariate analyses, the community data were Hellinger pretransformed. This transformation allowed us to compare communities using response data in the same format, which facilitates the analyses and homogenizes the interpretation of the results (Legendre & Gallagher, 2001).

#### Spatial structure of AM fungal communities

To investigate how AM fungal root colonization, richness, diversity and evenness varied across multiple spatial scales for both root and soil samples, we modelled each response variable as a function of the fixed effects ‘region’, ‘population’ (nested within ‘region’), and ‘subpopulation’ (nested within ‘population’). To compare OTU richness (observed OTU count), Shannon's diversity, and Pielou's evenness for root and soil, we accounted for differences in sequencing depth by using the residual values of linear models, in which the response variables were expressed as a function of the square root of the total sequence number per sample (Tedersoo *et al*., 2014; Bálint *et al*., 2015). Sampling efficacy was further assessed using the function *rarecurve* in the R‐package vegan (Oksanen *et al*., 2015; Fig. S2).

To test how AM fungal communities varied across multiple spatial scales we used both presence‐absence and absolute count data. Communities were analysed by permutation‐based ANOVA using the function *adonis* in the R‐package vegan with the Euclidian dissimilarity index (Oksanen *et al*., 2015). To investigate if differences in sequencing depth influenced the results we added the square root of total sequences per sample to the models. Spatial variation of the AM fungal response variables (root colonization, species richness, diversity, evenness and community composition) was partitioned between the four hierarchical scales (neighbourhood, subpopulation, population and region) by dividing the sums of squares of each factor individually by the total sums of squares (Quinn & Keough, 2002). See Tables S1 and S2 for full ANOVA and permutation‐based ANOVA tables.

#### Environmental drivers of AM fungal communities

We started with assessing – akin to the analyses for the AM fungal community – the variation in the abiotic and biotic factors ascribed to spatial structure at each hierarchical scale by modelling each response variable as a function of the fixed effects ‘region’, ‘population’ (nested within ‘region’) and ‘subpopulation’ (nested within ‘population’). As soil nutrients and climate were assessed at the subpopulation scale, we did not include the factor ‘subpopulation’ in these models, and for those response variables, the residuals thus represent the pooled within‐population variation. For the nonAM fungal root‐associated diversity indices, we accounted for differences in sequencing depth by using the residuals of linear models with the square root of the total sequence number per sample as the explaining variable (Tedersoo *et al*., 2014).

To test how individual abiotic and biotic environmental factors influenced AM fungal richness, diversity, and evenness, we used multiple regression models. In order to avoid collinearity, we excluded a few of the predictor variables, and thereby modelled each of the response variables as a function of soil chemical characteristics (pH, P, NH_4_ and NO_3_), bioclimatic variables (mean below‐ and above‐ground temperatures in the growing and nongrowing seasons, mean annual relative humidity, and soil moisture), neighbouring plant richness, and nonAM fungal root‐associated richness. To determine how the environment influenced the composition of soil and root‐associated AM fungal communities, we used partial canonical redundancy analysis (partial RDA; Legendre & Legendre, 2012, section 11.1) using all environmental variables measured. We controlled for spatial structure using Moran's eigenvector maps (MEMs; see next section). The significance of each RDA axis was tested using the function *anova.cca* in the R‐package vegan.

In order to test if AM fungal community composition was related to the composition of the neighbouring plant community and to the composition of the nonAM root‐associated fungi we used Procrustes analysis and its associated test (Jackson, 1995) available in the R‐package vegan through the *protest* function.

#### Relative importance of spatial structure and environment in shaping AM fungal communities

We determined the importance of spatial structure and environmental factors, as well as their overlapping effect, in explaining variation in AM fungal colonization, richness, diversity, evenness, and community composition. To assess the spatial structure of the AM fungal community, we used MEMs, which were constructed through the diagonalization of a connection matrix weighted by the inverse of the Euclidean distance among samples (Fig. S3; Dray *et al*., 2006), resulting in both positive and negative MEMs (Borcard & Legendre, 2002; Dray *et al*., 2006). Following this, we constructed a separate set of MEMs for the root (*n* = 104) and soil (*n* = 96) AM fungal communities. The significance of the autocorrelation measured by each MEM was tested using the function *moran.randtest* in the R‐package adespatial (Dray *et al*., 2017), allowing identification of the MEMs that described the spatial pattern significantly (*P* < 0.05) better than randomly expected.

To choose the MEMs that were important in structuring the fungal community, we performed forward selection independently on the root and soil data, using the approach proposed by Blanchet *et al*. (2008). Only positively autocorrelated MEMs were selected and used for all other analyses.

For certain samples, missing values for specific environmental variables were interpolated by multiple regression with the other environmental variables as predictors. All interpolated values were well within the range of measured values.

Variation partitioning was carried out using the *varpart* function in the R‐package vegan, using the selected MEMs and the environmental variables. Through partial RDA we used isolated independent fractions of the variation partitioning analysis and assessed their significance with permutation‐based ANOVA, using the *anova.cca* function in the R‐package vegan. We used adjusted *R* ^2^ to evaluate the contribution of each fraction, as the adjusted *R* ^2^ has been recommended by Peres‐Neto *et al*. (2006) in variation partitioning using RDA models with Hellinger‐transformed data, and as it allowed for a more direct comparison between root and soil AM fungal communities, for which models were built on different numbers of explanatory variables. We note that a negative adjusted *R* ^2^ can be interpreted as an adjusted *R* ^2^ of 0 (Peres‐Neto *et al*., 2006).

#### Variation between AM fungal communities in roots and soil

To investigate how AM fungal richness, diversity, and evenness differed between root and soil communities, each response variable was modelled as a function of ‘sample type’ as a fixed effect and ‘plant individual’ as a random effect. In order to validate that differences in OTU richness between the roots and root‐associated soil were not an artefact of the differences in sequence number between root and root‐associated soil samples, rarefied OTU richness, based on resampling to the mean number of soil reads per sample (*n* = 4305; Fig. S2), was calculated using the function *rarefy* in the package vegan (Oksanen *et al*., 2015), which is based on Hurlbert (1971) and Heck *et al*. (1975). To test for differences in community composition between root and root‐associated soil we used linear discriminant analysis with the function *lda* in the R‐package mass (Venables & Ripley, 2002). We tested for significant differences between the two fungal communities using a chi‐squared test.

## Results

**Figure 2:**
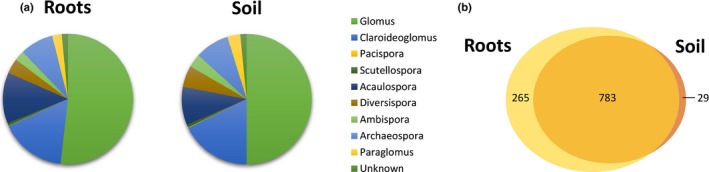
An overview of the fungal community in the roots and root‐associated soil of *Plantago lanceolata*. (a) The proportion of arbuscular mycorrhizal (AM) fungal operational taxonomic units (OTUs) within each genus in the roots and the root‐associated soil. (b) Venn diagram illustrating the number of OTUs found exclusively in roots, found in both roots and root‐associated soil, and found exclusively in the root‐associated soil.

We identified 1077 AM fungal OTUs from a total of 2 727 662 sequences. From these, 2 314 361 sequences were assigned to 1048 OTUs in the roots of *P. lanceolata* (*n* = 104 root samples) and 413 301 sequences were assigned to 812 OTUs in the surrounding soil (*n* = 96 soil samples). In total, 98.5% of all AM fungal OTUs were identified to genus level (Fig. 2). A majority (90%) of the OTUs were found in < 25% of all samples, while six OTUs were found in > 75% of all samples. These six very common OTUs all belonged to the genus *Glomus*.

### Spatial structure of AM fungal communities

**Figure 3:**
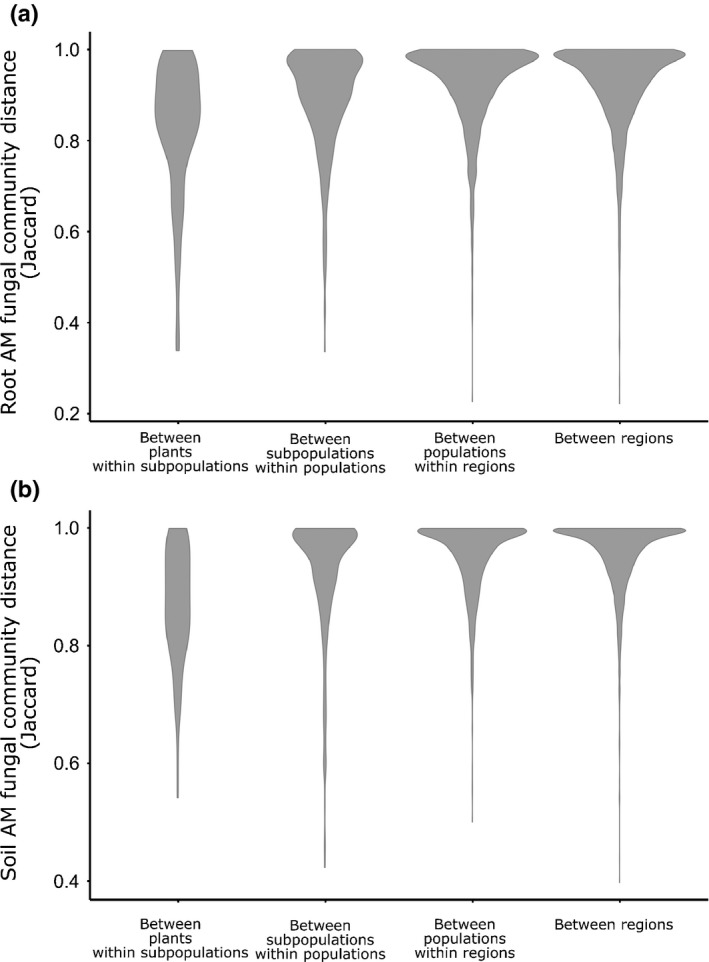
Violin plots showing the pairwise Jaccard distance between arbuscular mycorrhizal (AM) fungal communities in root (a) and soil (b) samples of *Plantago lanceolata*.

Arbuscular mycorrhizal fungal communities differed significantly between subpopulations and populations for most community descriptors (Table 2). Community composition in both roots and soil was significantly structured at all spatial scales, with small but significant variation also at the regional level (Table 2; Fig. 3). For all community descriptors, the majority of variation was found at the smallest spatial scale (i.e. between neighbouring plants), with variation decreasing at increasing spatial scales, a pattern consistent for both root and soil samples (Table 2). Differences in sequencing depth had a significant but small effect, explaining 1–2% of the variation in community composition (Table S2).

**Table 2:** Percentage of variation explained at each spatial scale for root colonization and community descriptors in both root and root‐associated soil of *Plantago lanceolata* using either ANOVA (root colonization, richness, diversity and evenness) or PERMANOVA (community composition for both the presence‐absence and abundance of operational taxonomic units (OTUs))

	Root colonization	Richness		Diversity		Evenness		Community composition presence‐absence		Community composition abundance	
		Root	Soil	Root	Soil	Root	Soil	Root	Soil	Root	Soil
Region	1.0	3.9	0.1	1.2	0.5	1.5	0.1	**4.0**	**3.5**	**4.4**	**4.4**
Population	**14.7**	11.2	**28.8**	**22.7**	**29.4**	**18.6**	**29.8**	**14.6**	**14.1**	**20.7**	**18.5**
Subpopulation	**37.9**	22.7	16.2	27.7	**27.5**	**30.5**	23.1	**29.0**	**28.3**	**35.1**	**31.2**
Plant neighbourhood^a^	46.4	62.1	54.8	48.4	42.5	49.4	47.1	52.5	54.1	39.9	46.0

Significant estimates (*P* < 0.05) are shown in bold.

a No significance levels were calculated for plant neighbourhood as these estimates are based on the residual variation.

### Environmental drivers of AM fungal communities

The soil chemical characteristics and climatic variables differed in their variation across the three measured spatial scales (subpopulation, population and region): NO_3_ and total N varied mostly among regions; NH_4_, below‐ground and above‐ground temperatures and relative humidities in the growing season varied mostly among populations; and pH and above‐ground temperatures in the nongrowing season varied mostly within populations (Table S3; Fig. S4). Most variation in AM fungal community descriptors was found at the smallest spatial scale measured (among neighbouring plants). Similar to this, vegetation and nonAM root‐associated fungi varied mostly at the smaller spatial scales measured (at the plant neighbourhood and subpopulation scales) with decreasing variation at the population and regional scales (Table S3).

**Figure 4:**
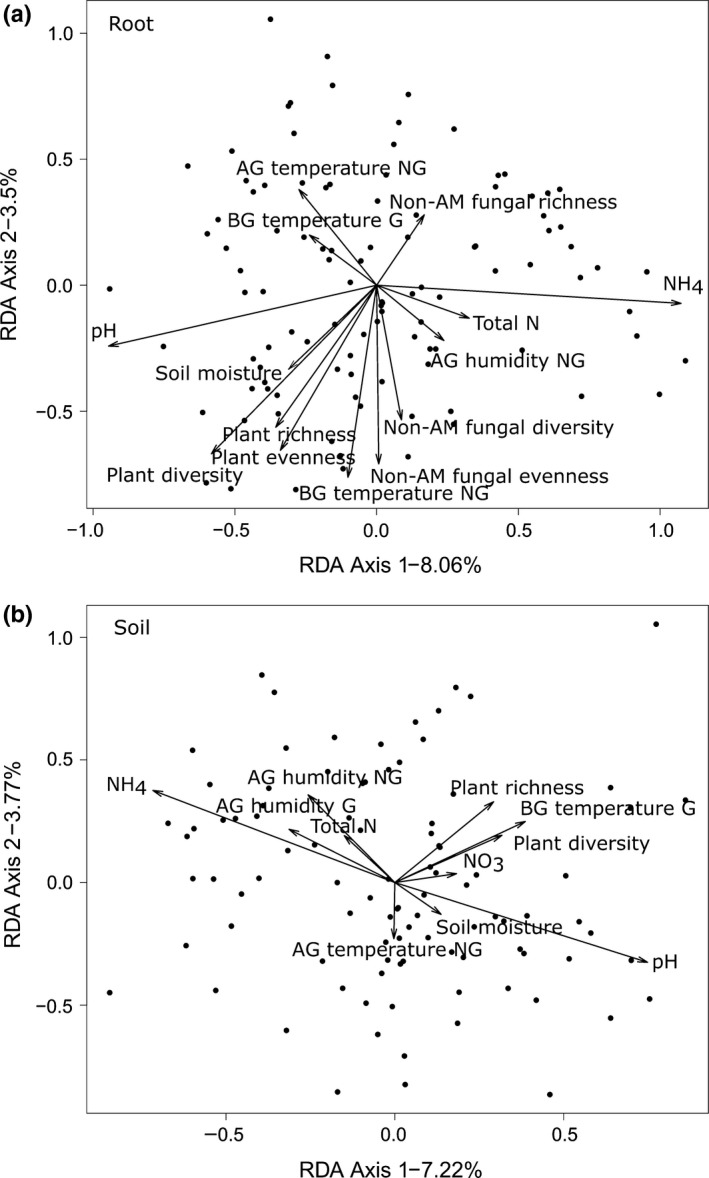
Partial canonical redundancy analysis (partial RDA) ordination plots for arbuscular mycorrhizal (AM) fungal communities in root (a) and root‐associated soil (b) of *Plantago lanceolata* where the main environmental variables are shown. All environmental variables with arrows very close to the centre were removed for visual clarity and because no interpretation could be gained from them. For each analysis, the Moran's eigenvector maps were used to control for spatial structure. Each point represents an AM fungal community found within a single sample, while vectors show the main environmental drivers. A correlation scaling was used to draw each ordination plot so that angles between variables could be interpreted directly. AG, above ground; BG, below ground; G, growing season; NG, nongrowing season.

Descriptors of the AM fungal community were differently related to the abiotic and biotic variables (Table S4). Colonization of roots by AM fungi increased whereas AM fungal evenness decreased with increasing soil moisture. Strikingly, AM fungal richness was not affected by any of the environmental variables. AM fungal diversity and evenness in the roots were negatively affected by increasing temperatures below ground in the nongrowing season and above ground in the growing season. AM fungal diversity in the root‐associated soil and AM fungal evenness in both root and root‐associated soil increased under less acidic conditions. Accordingly, AM fungal community composition in both roots and soil appeared to be strongly influenced by pH and NH_4,_ with opposing effects (Fig. 4). Furthermore, soil moisture, temperature, and plant and nonAM fungal diversity indices were related to the composition of AM fungal communities in roots (Fig. 4a), whereas humidity, temperature and plant diversity indices correlated with AM fungal community composition in the root‐associated soil (Fig. 4b).

Arbuscular mycorrhizal fungal communities in both roots and root‐associated soil were correlated to the neighbouring plant communities (*r* = 0.53, *n* = 104, *P* = 0.001; and *r *= 0.60, *n* = 96, *P* = 0.001, respectively) and to community composition of the nonAM root‐associated fungi (*r* = 0.73, *n* = 104, *P* = 0.001; *r* = 0.80, *n* = 96, *P* = 0.001, respectively).

### Relative importance of spatial structure and environment in shaping AM fungal communities

**Figure 5:**
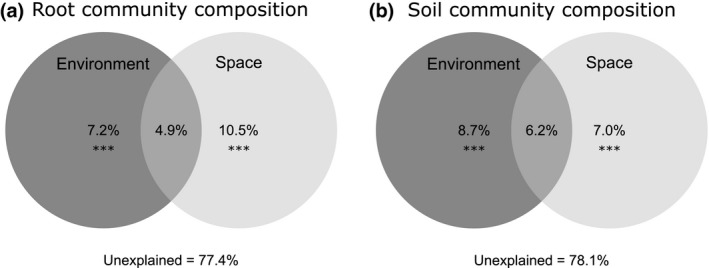
The relative importance of space and the environment in structuring the arbuscular mycorrhizal (AM) fungal community composition in the roots (a) and the root‐associated soil (b) of *Plantago lanceolata*. All values are presented using the adjusted coefficient of determination (*R* ^2^_adjusted_). The variation is partitioned into four fractions: purely environmental variation, purely spatial variation, both environmental and spatial variation, and unexplained variation (residuals). Asterisks indicate that a significant amount of variation is explained by the given fraction (***, *P* < 0.001).

Spatial structure and environmental variables explained a similar amount of variation in the majority of community descriptors (7.0–22.5% and 7.2–25.1%, respectively; Figs 4, S5). However, spatial structure had a significant influence on most aspects of the AM fungal communities, whereas environmental variables were only significant in relation to community composition, colonization and diversity in soil (Table S4). Combined spatial and environmental variation explained 0–13.4% of the variation in AM fungal community variables. Despite the use of a large set of spatial and environmental variables in the analyses, a major fraction of the variation in the AM fungal community remained unexplained (Figs 5, S5).

### Variation between AM fungal communities in roots and soil

Roots hosted higher overall richness of AM fungal OTUs than the root‐associated soil (mean ± SD per sample: 120 ± 70 and 65 ± 45, respectively), as confirmed by a significantly higher richness in roots when using both residual and rarefied richness (*χ* ^2^ = 12.46, d.f. = 1, *P* < 0.001 and *χ* ^2^ = 34.45, d.f. = 1, *P* < 0.001, respectively). Evenness was lower in roots than in root‐associated soil (*χ* ^2^ = 27.56, d.f. = 1, *P* < 0.001). Most OTUs were found in both roots and the root‐associated soil, although some OTUs were found exclusively in either roots (25%) or root‐associated soil (3%; Fig. 2b). While there was a large overlap in OTUs and a fairly equal representation of species within genera (Fig. 2), AM fungal communities differed significantly between roots and root‐associated soil (*χ* ^2^ = 153.41, d.f. = 1, *P *< 0.001). AM fungal communities in the roots and root‐associated soil were more similar but more variable at small spatial scales, whereas communities were increasingly dissimilar at larger spatial scales (Fig. S6).

## Discussion

In this study we investigated the spatial variation and drivers of AM fungal communities associated with a wild plant species in a fragmented landscape. We found that AM fungal communities were already highly dissimilar among neighbouring plants, even though there was considerable variation in similarity at this small spatial scale. AM fungal communities were increasingly, and more consistently, dissimilar at larger spatial scales. This pattern matched the spatial variation in some of the key abiotic (pH) and biotic (plant neighbourhood and nonAM fungal community) predictors of AM fungal community structure, which also varied mostly within populations. Spatial structure and environmental parameters were equally important predictors of AM fungal communities, and largely explained different parts of the variation. Finally, even though there was a large overlap in AM fungal OTUs between root and root‐associated soil communities, community composition in root and soil differed, and we found a higher species richness in the roots.

### Spatial structure of AM fungal communities

Communities of AM fungi in both the roots and root‐associated soil were already found to vary strongly among neighbouring *P. lanceolata* plants, with substantial additional variation among plant subpopulations. There was less, but still significant, spatial structuring in the AM fungal communities associated with the focal plant species at scales ranging from a few to tens of kilometres (i.e. among populations or regions). Chaudhary *et al*. (2014) also observed spatial structure in AM fungal abundance and diversity at different scales in a semiarid region of southern Utah (Table 1). Similarly, we found that AM fungal communities furthest apart were consistently highly dissimilar, while similarity between closely located samples was higher and more variable. However, in contrast to our findings, Chaudhary *et al*. (2014) found most variation at the larger spatial scales (between regions *c*. 50–100 km apart). This discrepancy may be a result of differences in methodology between the two studies, such as sampling of different habitats (small dry meadows in an agricultural landscape vs a semiarid shrub land) or the investigation of the community in roots and root‐associated soil from single plants in the present study, as compared with abundance and diversity measures per microsite (1 m^2^) in Chaudhary *et al*. (2014).

Most other studies on the spatial distribution of AM fungi have focused on either very small or very large scales. In agreement with our findings of large variation in the AM fungal community at small spatial scales, several studies have found large variation at the submetre and metre scales (Whitcomb & Stutz, 2007; Mummey & Rillig, 2008). However, it is important to note that these studies sampled AM fungal communities associated with mixed vegetation. Hence, the small‐scale spatial variation in the AM fungal community structure may have been a result of specificity in plant–fungal associations rather than spatial variation of the AM fungal community associated with a single plant species. At the larger regional scale (25 km), communities of AM fungi in cultivated maize fields were found to become increasingly dissimilar with increasing distance (Lekberg *et al*., 2007). Studies at the global scale have similarly found that AM fungal communities become more dissimilar with increasing geographic distance (Kivlin *et al*., 2011; Davison *et al*., 2015), which is in general agreement with our results.

### Drivers of AM fungal communities

The abiotic and biotic variables pH, neighbouring vegetation and nonAM root‐associated fungi were spatially structured in a similar way as the AM fungi, being highly variable at the smallest spatial scales at which they were measured (within populations), and much less variable across larger spatial scales (among populations and among regions). In comparison, other measured abiotic factors (such as NO_3_, total N, temperature, and relative humidity) varied primarily among populations and regions. Overall, spatial structure and environmental factors explained *c*. 9% and 11%, respectively, of the variation in AM fungal community structure.

Our exploratory analysis of the environmental drivers of AM fungal communities pinpoints several important abiotic and biotic factors. Like several other studies, we found that pH and nitrogen availability strongly influenced AM fungal community composition (Jumpponen *et al*., 2005; Lekberg *et al*., 2007, 2011; Dumbrell *et al*., 2010; van Diepen *et al*., 2011). We also found that higher temperatures led to a decrease in diversity and evenness of AM fungi and influenced the AM fungal community composition. Given projected increases in temperatures (European Environment Agency, 2017) and the observation that warmer temperatures seem to favour potential dominants in the AM fungal community, these findings suggest that we may expect less diverse AM fungal communities in the future.

Our findings show a strong relationship between AM fungal communities and plant and nonAM fungal communities. Evidence for a connection between communities of plant and AM fungi have been found both in experimental studies (Stampe & Daehler, 2003; Johnson *et al*., 2004) and in natural systems (Öpik *et al*., 2006; Kivlin *et al*., 2011). Such a link may indicate both specificity between AM fungi and plants (Kivlin *et al*., 2011; Velázquez *et al*., 2013; Rodríguez‐Echeverría *et al*., 2017) and a similar response of plants and AM fungi to the abiotic environment. While the relationship between AM fungal and plant communities has been studied frequently, the relationship between the AM and nonAM fungal communities has been studied to a much lesser extent. In this study, we found a strong relationship between AM and nonAM fungal root‐associated communities. The relationship between these distinct fungal groups may have two nonmutually exclusive explanations: AM and nonAM root‐associated fungi respond to the same abiotic drivers; or positive and negative interactions between AM and nonAM fungi result in predictable co‐occurrence patterns (Larimer *et al*., 2012).

The high variation at the small spatial scale reported here may be a result of both the expected low dispersal abilities of AM fungi over short distances, in addition to competitive interactions between AM fungal species and priority effects (Fukami *et al*., 2010). Lekberg *et al*. (2012) found that mainly stochastic processes were shaping AM fungal communities at the local scale. Similarly, reanalysing the data of Davison *et al*. (2015), Powell & Bennett (2016) found that AM fungal community composition was highly unpredictable within similar environments. In the current study we found that *c*. 80% of the variation in AM fungal community composition could not be explained by spatial structure or the environment. This seems to match other studies on plant‐associated fungal communities, which generally report a relatively large fraction (*c*. 35–80%) of unexplained variation (e.g. Lekberg *et al*., 2007; Sterkenburg *et al*., 2015; Horn *et al*., 2017). Such unexplained variation is probably a result of unmeasured environmental variables, species interactions and stochastic processes.

### Variation between AM fungal communities in roots and soil

We found that the composition of AM fungal communities was largely similar between roots and the root‐associated soil, with roots and soil hosting similar proportions of AM fungal genera and sharing most of the OTUs. However, roots hosted an overall higher richness than the root‐associated soil. This is in contrast to some studies which have suggested that the soil contains a species pool of AM fungi from which the plants can be colonized (Davison *et al*., 2011). While many studies have found higher AM fungal richness in soil than in roots (Bainard *et al*., 2011; Martínez‐García *et al*., 2011; Varela‐Cervero *et al*., 2015), others have found equal or lower richness in the soil (Verbruggen *et al*., 2012; Saks *et al*., 2014). While these findings may reflect real ecological patterns, the patterns could also be ascribed to methodological differences. Methodological differences in sequencing depth between root and soil samples may be a result of biological characteristics of AM fungi: AM fungi generally contain lower densities of biomass in soil than in roots (Olsson *et al*., 2010), and the distribution of nuclei in the extraradical mycelium can be highly variable (Gamper *et al*., 2008). Moreover, divergent composition of AM fungal communities in roots and root‐associated soil may be caused by differences among taxa in their relative abundance of intra‐ or extraradical structures (Hart *et al*., 2001) and seasonality of spore production (Bever *et al*., 2001; Jansa *et al*., 2002).

### Conclusion

Arbuscular mycorrhizal fungal communities differed strongly among neighbouring plants and among plant subpopulations, but there was also strong variation in dissimilarity at these small spatial scales. At the larger spatial scales, AM fungal communities were more consistently dissimilar. Spatial structure and environmental predictors explained equal parts of variation in AM fungal communities. For example, pH, vegetation and nonAM root‐associated fungi, which were just like the AM fungal community most variable at small spatial scales, were correlated with the structure of the AM fungal community. While pH may affect the AM fungal community composition directly, the question of ‘who drives who’ is more difficult to answer for the biotic factors: while we focused on predictors of the AM fungal community, it may well be that the AM fungi themselves contribute to shaping the nonAM fungal and plant communities. A promising avenue for future research would be to conduct experimental studies to disentangle the causal drivers and reciprocal interactions among AM fungi, nonAM fungi, and the neighbouring plant community. Given the small scale at which the AM fungal communities vary, a promising avenue for future research may be to measure the abiotic and biotic environments at a finer scale than in the current study, and link this fine‐scale variation in the environment to the AM fungal community structure. Such studies may also target within‐plant variation in the AM fungal community. Overall, our findings contribute fundamental knowledge by highlighting the large variation in AM fungal community structure at small spatial scales, which may in turn add to the local diversity of both plants and plant‐associated organisms. From an applied perspective, understanding the distribution and drivers of the AM fungi may be important for conservation and agroecological management.

## Author contributions

P.U.R., A.J.M.T., A.F.A. and L.W.H. conceived and designed the experiment. P.U.R. conducted the empirical work. P.U.R. and L.W.H. conducted the molecular work, and P.U.R., L.W.H. and B.D.L. performed the bioinformatic analyses. P.U.R., A.J.M.T. and F.G.B. analysed the data. P.U.R. wrote the first draft, and all authors contributed to the final manuscript.

## Supporting information

Please note: Wiley Blackwell are not responsible for the content or functionality of any Supporting Information supplied by the authors. Any queries (other than missing material) should be directed to the *New Phytologist* Central Office.

**Fig. S1** The proportion of nonAM root‐associated fungal classes within the roots of *Plantago lanceolata*.
**Fig. S2** Rarefaction curves for AM fungi in roots and root‐associated soil.
**Fig. S3** Connection diagram of sampled *Plantago lanceolata* individuals in the Åland Islands in the Baltic Sea, southwestern Finland.
**Fig. S4** Variation of abiotic and biotic factors for each population.
**Fig. S5** Variation partitioning explaining AM fungal community descriptors.
**Fig. S6** Violin plot showing the distance between AM fungal communities in root and root‐associated soil for each hierarchical spatial scale.
**Table S1** ANOVA table for partitioning of variance for each hierarchical spatial scale
**Table S2** PERMANOVA table for partitioning of variance for each hierarchical spatial scale
**Table S3** Percentage of variation explained at each spatial scale for soil nutrients, bioclimatic variables, vegetation and nonAM root‐associated fungi at the regional, population, and subpopulation levels.
**Table S4** *P*‐values for the impact of environmental variables on the arbuscular mycorrhizal fungal root colonization and diversity indices in both root and root‐associated soil
**Methods S1** Full description of molecular methods and bioinformatics.
