# Supplementary material for: Multiscale patterns and drivers of arbuscular mycorrhizal fungal communities in the roots and root‐associated soil of a wild perennial herb
Source: New Phytol. 2018 Mar 24;220(4):1248–61. doi: 10.1111/nph.15088 (PMC6282561; doi:10.1111/nph.15088)
Supplement: Supplementary file 1 — Fig. S1 The proportion of nonAM root‐associated fungal classes within the roots of Plantago lanceolata. Fig. S2 Rarefaction curves for AM fungi in roots and root‐associated soil. Fig. S3 Connection diagram of sampled Plantago lanceolata individuals in the Åland Islands in the Baltic Sea, southwestern Finland. Fig. S4 Variation of abiotic and biotic factors for each population. Fig. S5 Variation partitioning explaining AM fungal community descriptors. Fig. S6 Violin plot showing the distance between AM fungal communities in root and root‐associated soil for each hierarchical spatial scale. Table S1 ANOVA table for partitioning of variance for each hierarchical spatial scale Table S2 PERMANOVA table for partitioning of variance for each hierarchical spatial scale Table S3 Percentage of variation explained at each spatial scale for soil nutrients, bioclimatic variables, vegetation and nonAM root‐associated fungi at the regional, population, and subpopulation levels. Table S4 P‐values for the impact of environmental variables on the arbuscular mycorrhizal fungal root colonization and diversity indices in both root and root‐associated soil Methods S1 Full description of molecular methods and bioinformatics. [file NPH-220-1248-s001.pdf]

New Phytologist **Supporting Information**

Article title: **Multiscale patterns and drivers of AM fungal communities in the roots and root-associated soil of a wild perennial herb**

Authors: Pil U. Rasmussen, Luisa W. Hugerth, F. Guillaume Blanchet, Anders F. Andersson, Björn D. Lindahl, and Ayco J. M. Tack

Article acceptance date: 11 February 2018

The following Supporting Information is available for this article:

Methods S1 Full description of molecular methods and bioinformatics.

Fig. S1 The proportion of non-AM root-associated fungal classes within in the roots of *Plantago lanceolata.*

Fig. S2 Rarefaction curves for AM fungi in roots and root-associated soil.

Fig. S3 Connection diagram of sampled *Plantago lanceolata* individuals in the Åland Islands in the Baltic Sea, SW Finland.

Fig. S4 Variation of abiotic and biotic factors for each population.

Fig. S5 Variation partitioning explaining AM fungal community descriptors.

Fig. S6 Violin plot showing the distance between AM fungal communities in root and root-associated soil for each hierarchical spatial scale.

Table S1 ANOVA table for partitioning of variance for each hierarchical spatial scale.

Table S2 PERMANOVA table for partitioning of variance for each hierarchical spatial scale.

Table S3 Percentage of variation explained at each spatial scale for soil nutrients, bioclimatic variables, vegetation and non-AM root-associated fungi at the regional, population, and subpopulation level.

Table S4 P-values for the impact of environmental variables on the arbuscular mycorrhizal fungal root colonisation and diversity indices in both root and root-associated soil.

Methods S1

Full description of molecular methods

SEQUENCING OF ARBUSCULAR MYCORRHIZAL FUNGI

To assess the AM fungal community composition within roots and root-associated soil, we used the primers NS31 and AML2 that target a *c.* 560-bp central fragment of the SSU rRNA gene in the Glomeromycota (Simon *et al.*, 1992; Lee *et al.*, 2008). These primers have often been used to assess the community composition of AM fungi (Öpik *et al.*, 2010; Davison *et al.*, 2015).

PCR amplification was carried out in two steps. For the first PCR reaction the mixture with a total volume of 30 µL consisted of 15 µL Kapa HiFi Mastermix (Kapa Biosystems), 10 µL H_2O, 1.5 µL of each primer (5 nmol/µL), and 2 µL of 4 ng/µL DNA template. PCR was conducted on the MasterCycler Pro S (Eppendorf). The primers in this first reaction consisted of adaptor + **primer**, giving the forward primer 5'– TCGTCGGCAGCGTCAGATGTGTATAAGAGACAG**TTGGAGGGCAAGTCTGGTGCC** – 3' and reverse primer 5'– GTCTCGTGGGCTCGGAGATGTGTATAAGAGACAG**GAACCCAAACACTTTGGTTTCC** – 3'. Cycling conditions were 95°C for 5 min, 98°C for 1 min, 25 cycles of 98°C for 40 sec, 58°C for 40 sec, and 72°C for 15 sec, followed by a final elongation step of 72°C for 5 min. Samples were cleaned as described by Lundin et al. (2010) with 14.4% PEG 6000, reducing the sample volume to 15 µL. To this volume, 20 µL Kapa HiFi Mastermix and 2.5 µL of each primer (10 nmol/µL) were added, giving a total volume of 40 µL for the second PCR reaction. The primers for the second PCR reaction consisted of Illumina handle + barcode + adaptor, giving the primers 5' AATGATACGGCGACCACCGAGATCTACAC-X_8-TCGTCGGCAGCGTC and 5'-CAAGCAGAAGACGGCATACGAGAT-X_8-GTCTCGTGGGCTCGG - 3'. X_8 denotes added tags consisting of an 8 bp DNA code giving each sample a unique barcode. Reaction conditions for the second PCR were as described above, but with 11 cycles instead of 25. The product was cleaned again (as described above), and concentrations measured using the Qubit dsDNA BR assay kit (Life Technologies), after which all the samples were pooled in equal concentrations. The product was cleaned a final time (as described above) and sequenced at SciLifeLab/NGI (Solna, Sweden) on MiSeq (Illumina Inc.).

SEQUENCING OF NON-AM ROOT-ASSOCIATED FUNGI

To assess non-AM root-associated fungi we used primers targeting the internal transcribed spacer (ITS) region, which has been selected as the universal barcode for fungi (Schoch *et al.*, 2012).

Specifically, we used the forward primer fITS7 (Ihrmark *et al.*, 2012) and the reverse primer ITS4 (White *et al.*, 1990) that target a 250-450 bp fragment encompassing the entire ITS2 with flanking sequences in the 5.8 and LSU genes. We followed the protocol of Clemmensen et al. (2016). In short, PCR reactions were run in a volume of 50 µL, using an individually tagged primer mixture which consisted of 5 µM fITS7 (CX_8T-GTGARTCATCGAATCTTTG) and 3 µL ITS4 (CX_8T-CCTCCGCTTATTGATATGC). X_8 denotes the added tags consisting of an eight bp long individual DNA code, giving each sample a unique barcode that differed from others in at least 3 positions. PCR reactions were run in triplicates, and sample-specific cycling conditions were modified so that each sample gave medium strong bands on agarose gel. The final amplicon pool was sequenced at SciLifeLab/NGI (Uppsala, Sweden) on a PacBio RS II system (Pacific Biosciences, California, USA).

Bioinformatics

AM FUNGI - Raw sequences were quality trimmed using Cutadapt (Martin, 2011) to remove 3' bases with Phred-score below 15 and 5' primer sequences, and read pairs that did not contain both primer sequences were discarded. All forward reads were then trimmed to 275 bp and reverse reads to 260 bp, to allow concatenation of forward and reverse reads (since merging was not possible, due to the amplicon length). Reads that did not reach these length cut-offs were discarded. This left a total of 10,188,003 reads. Vsearch (Rognes *et al.*, 2016) was used to map sequences to the MaarjAM database (Öpik *et al.*, 2010) and sequences not belonging to the Glomeromycota (< 90% identity) were removed (c. 60%). The remaining sequences were clustered based on 99% similarity also using Vsearch. This cut-off has previously been used in studies on AM fungal biogeographical patterns (Kivlin *et al.*, 2011; but see Bruns & Taylor, 2016 and Bruns *et al.*, 2017). From the final OTU table, rare OTUs (≤ 5 reads) were removed.

NON-AM ROOT-ASSOCIATED FUNGI – Reads obtained from the fungal ITS primers were analysed in the bioinformatics pipeline SCATA (scata.mykopat.slu.se; Ihrmark *et al.*, 2012). Sequences with a minimum mean quality < 20 or a minimum base quality < 3 were discarded, and reverse reads were reverse-complemented. Sequences were screened for both primers, requiring a 90% match, as well as sample tags, and non-matching sequences were discarded. Sequences were pairwise compared using USEARCH (Edgar, 2010), then clustered into operational taxonomic units (OTU) using single linkage clustering with 98.5% sequence similarity to next neighbour required in order to enter clusters. Indels were penalised equally as substitutions. This resulted in a total of 104,149 reads, clustering into 1247 OTUs. Sequences were obtained from 77 samples with an average of 1370 reads per sample. For further analyses we used a set of the 179 most common OTUs, making up 90% of the total number of reads (after removing plant and AM fungal OTUs). These OTUs were tentatively identified to the

species level using Species Hypotheses in the UNITE database (Kõljalg *et al.*, 2005; Abarenkov *et al.*, 2010).

References

Abarenkov K, Henrik Nilsson R, Larsson K-H, Alexander IJ, Eberhardt U, Erland S, Høiland K, Kjøller R, Larsson E, Pennanen T, *et al.* 2010. The UNITE database for molecular identification of fungi – recent updates and future perspectives. *New Phytologist* **186:** 281–285.

Bruns TD, Corradi N, Redecker D, Taylor JW, Öpik M. 2017. Glomeromycotina: what is a species and why should we care? *New Phytologist*: doi: 10.1111/nph.14913.

Bruns TD, Taylor JW. 2016. Comment on 'Global assessment of arbuscular mycorrhizal fungus diversity reveals very low endemism'. *Science* **351:** 826–826.

Clemmensen KE, Ihrmark K, Durling MB, Lindahl BD. 2016. Sample preparation for fungal community analysis by high-throughput sequencing of barcode amplicons. In: Martin F, Uroz S, eds. Methods in Molecular Biology, vol 1399. Microbial Environmental Genomics (MEG). Humana Press, New York, NY, 61–88.

Davison J, Moora M, Öpik M, Adholeya A, Ainsaar L, Bâ A, Burla S, Diedhiou AG, Hiiesalu I, Jairus T, *et al.* 2015. Global assessment of arbuscular mycorrhizal fungus diversity reveals very low endemism. *Science* **349:** 970–973.

Edgar RC. 2010. Search and clustering orders of magnitude faster than BLAST. *Bioinformatics* **26:** 2460–2461.

Ihrmark K, Bödeker ITM, Cruz-Martinez K, Friberg H, Kubartova A, Schenck J, Strid Y, Stenlid J, Brandström-Durling M, Clemmensen KE, *et al.* 2012. New primers to amplify the fungal ITS2 region - evaluation by 454-sequencing of artificial and natural communities. *FEMS Microbiology Ecology* **82:** 666–677.

Kivlin SN, Hawkes CV, Treseder KK. 2011. Global diversity and distribution of arbuscular mycorrhizal fungi. *Soil Biology and Biochemistry* **43:** 2294–2303.

Kõljalg U, Larsson K-H, Abarenkov K, Nilsson RH, Alexander IJ, Eberhardt U, Erland S, Høiland K, Kjøller R, Larsson E, *et al.* 2005. UNITE: a database providing web-based methods for the molecular identification of ectomycorrhizal fungi. *New Phytologist* **166:** 1063–1068.

Lee J, Lee S, Young JPW. 2008. Improved PCR primers for the detection and identification of arbuscular mycorrhizal fungi. *FEMS Microbiology Ecology* **65:** 339–349.

Lundin S, Stranneheim H, Pettersson E, Klevebring D, Lundeberg J. 2010. Increased throughput by parallelization of library preparation for massive sequencing. *PLoS ONE* **5:** e10029.

Martin M. 2011. Cutadapt removes adapter sequences from high-throughput sequencing reads. *EMBnet. journal* **17:** 10–12.

Öpik M, Vanatoa A, Vanatoa E, Moora M, Davison J, Kalwij JM, Reier ü., Zobel M. 2010. The online database MaarjAM reveals global and ecosystemic distribution patterns in arbuscular mycorrhizal fungi (Glomeromycota). *New Phytologist* **188:** 223–241.

Rognes T, Flouri T, Nichols B, Quince C, Mahé F. 2016. VSEARCH: a versatile open source tool for metagenomics. *PeerJ* **4:** e2584.

Schoch CL, Seifert KA, Huhndorf S, Robert V, Spouge JL, Levesque CA, Chen W, Bolchacova E, Voigt K, Crous PW, *et al.* 2012. Nuclear ribosomal internal transcribed spacer (ITS) region as a universal DNA barcode marker for Fungi. *Proceedings of the National Academy of Sciences* **109:** 6241–6246.

Simon L, Lalonde M, Bruns TD. 1992. Specific amplification of 18S fungal ribosomal genes from vesicular-arbuscular endomycorrhizal fungi colonizing roots. *Applied and Environmental Microbiology* **58:** 291–295.

White TJ, Bruns T, Lee SJWT, Taylor JW. 1990. Amplification and direct sequencing of fungal ribosomal RNA genes for phylogenetics. *PCR protocols: a guide to methods and applications* **18:** 315–322.

Fig. S1 The proportion of non-AM root-associated fungal classes within in the roots of *Plantago lanceolata*.

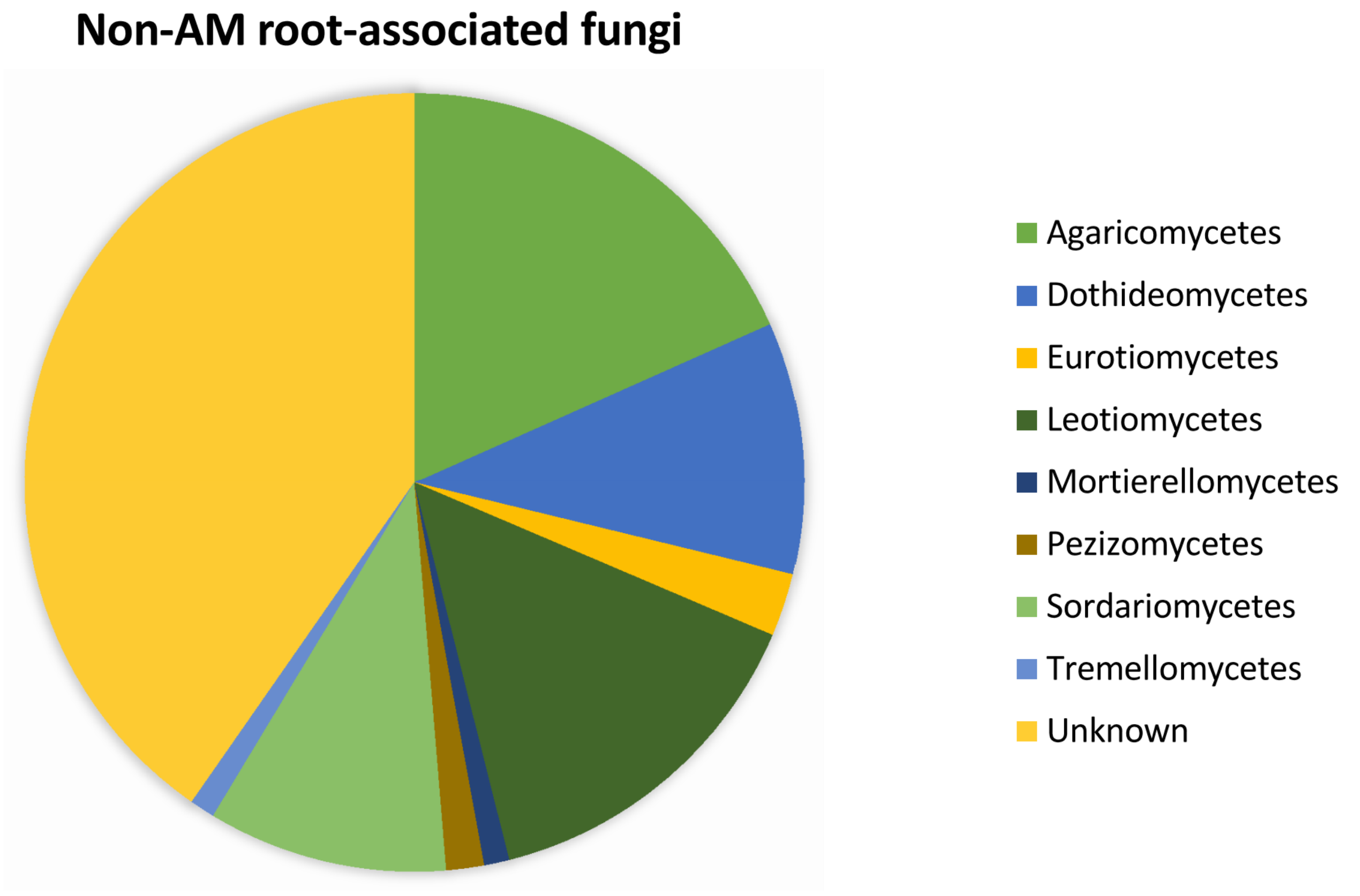

Fig. S2 Rarefaction curves for AM fungal (a) root and (b) root-associated soil samples. The vertical line shows the mean total sample reads for soil samples (n = 4305), which was used in order to evaluate sampling efficacy.

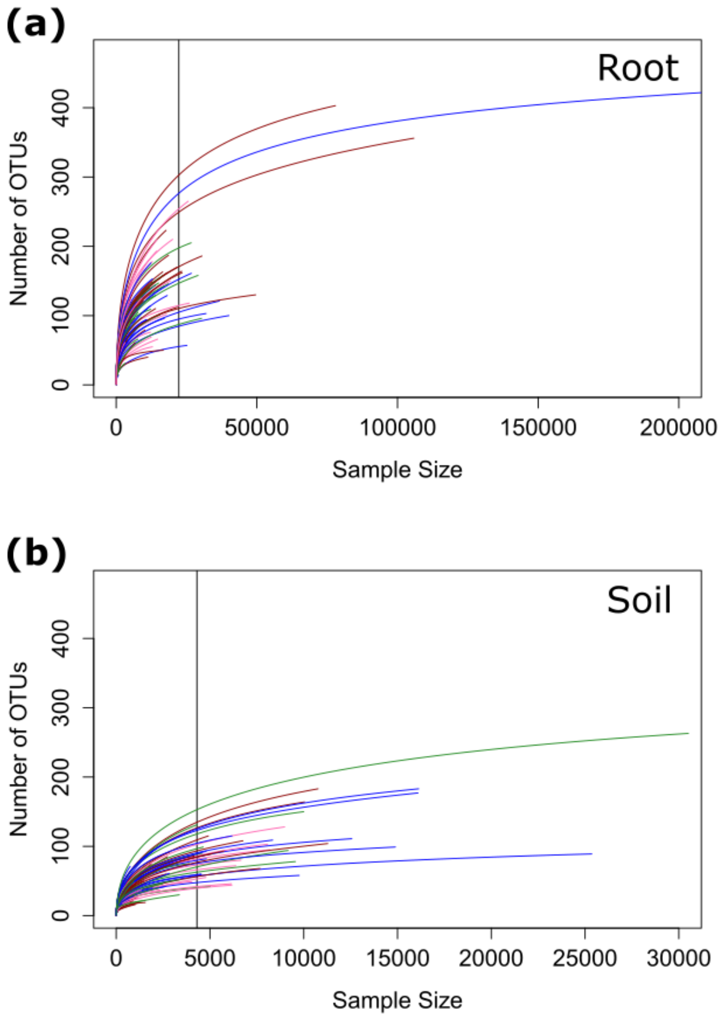

Fig. S3 Connection diagram linking the sampled *Plantago lanceolata* individuals of the Åland Islands located in the Baltic Sea, SW Finland. The connection diagram was used as a basis to construct Moran's eigenvector maps (MEMs). To construct the final MEMs, the weight applied to each link was the inverse of its length (i.e. Euclidean distance). Note that the plant neighbourhood scale is not illustrated in this figure. All plants within a subpopulation are considered neighbours of each other.

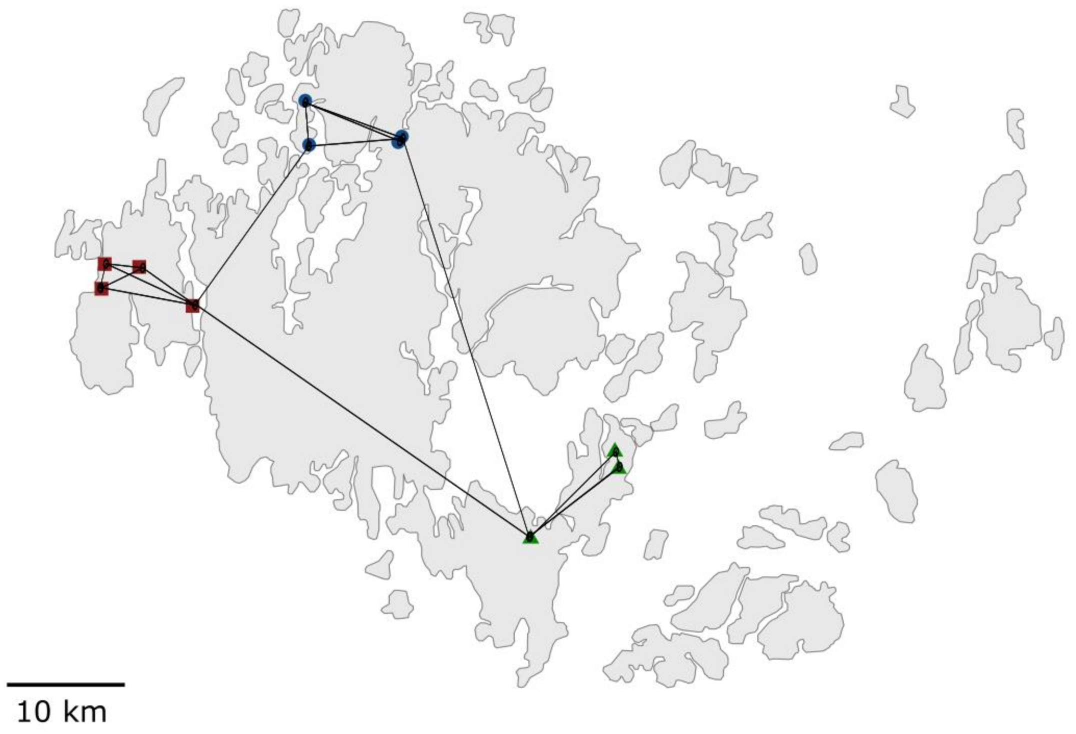

Fig. S4 Variation in the abiotic and biotic factors for each population. Colours and shapes represent samples from different regions (Eckerö, red circles; Geta, green triangles; Lumparland, blue squares). AG, aboveground; BG, belowground; G, growing season; NG, non-growing season.

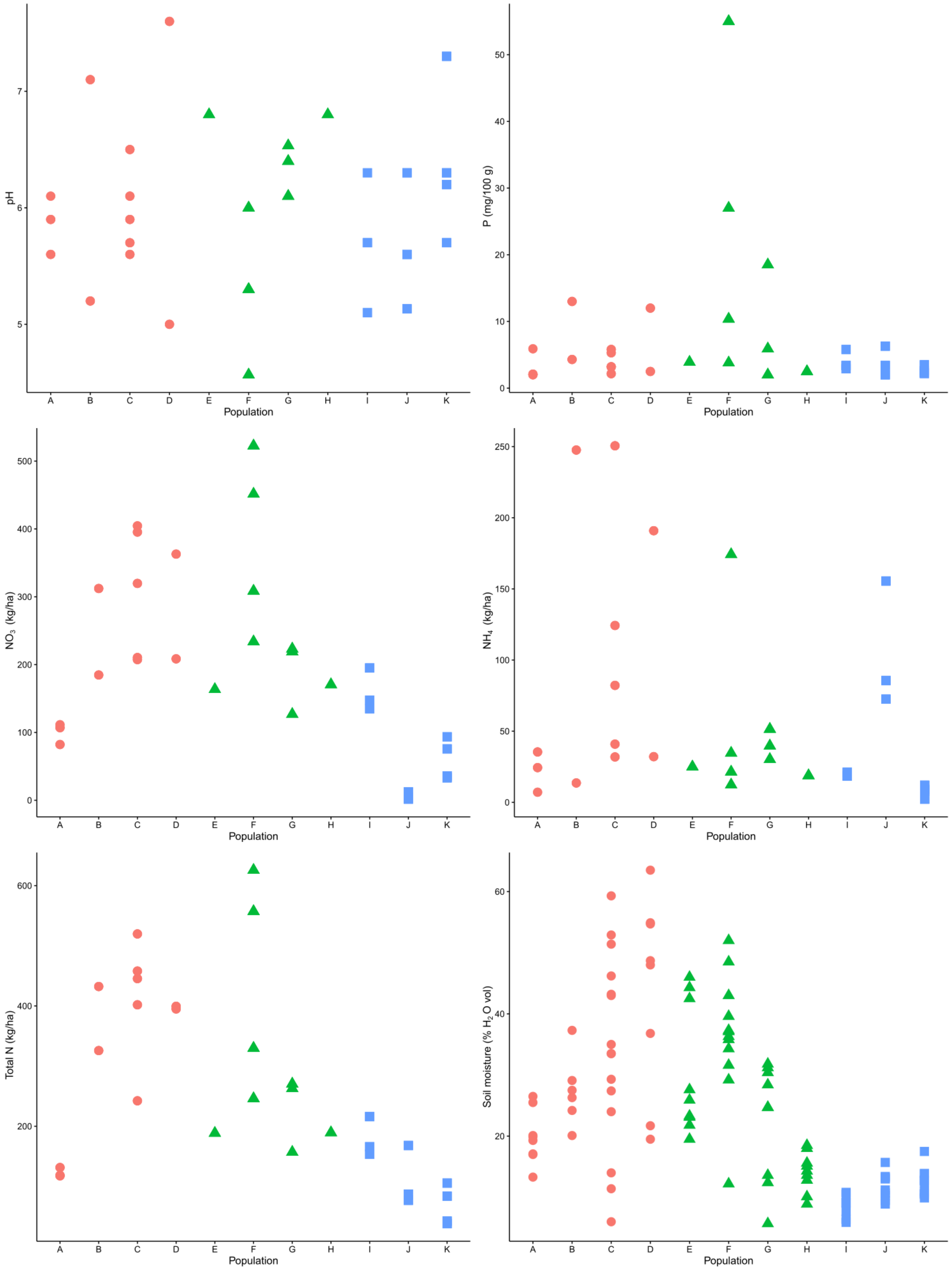

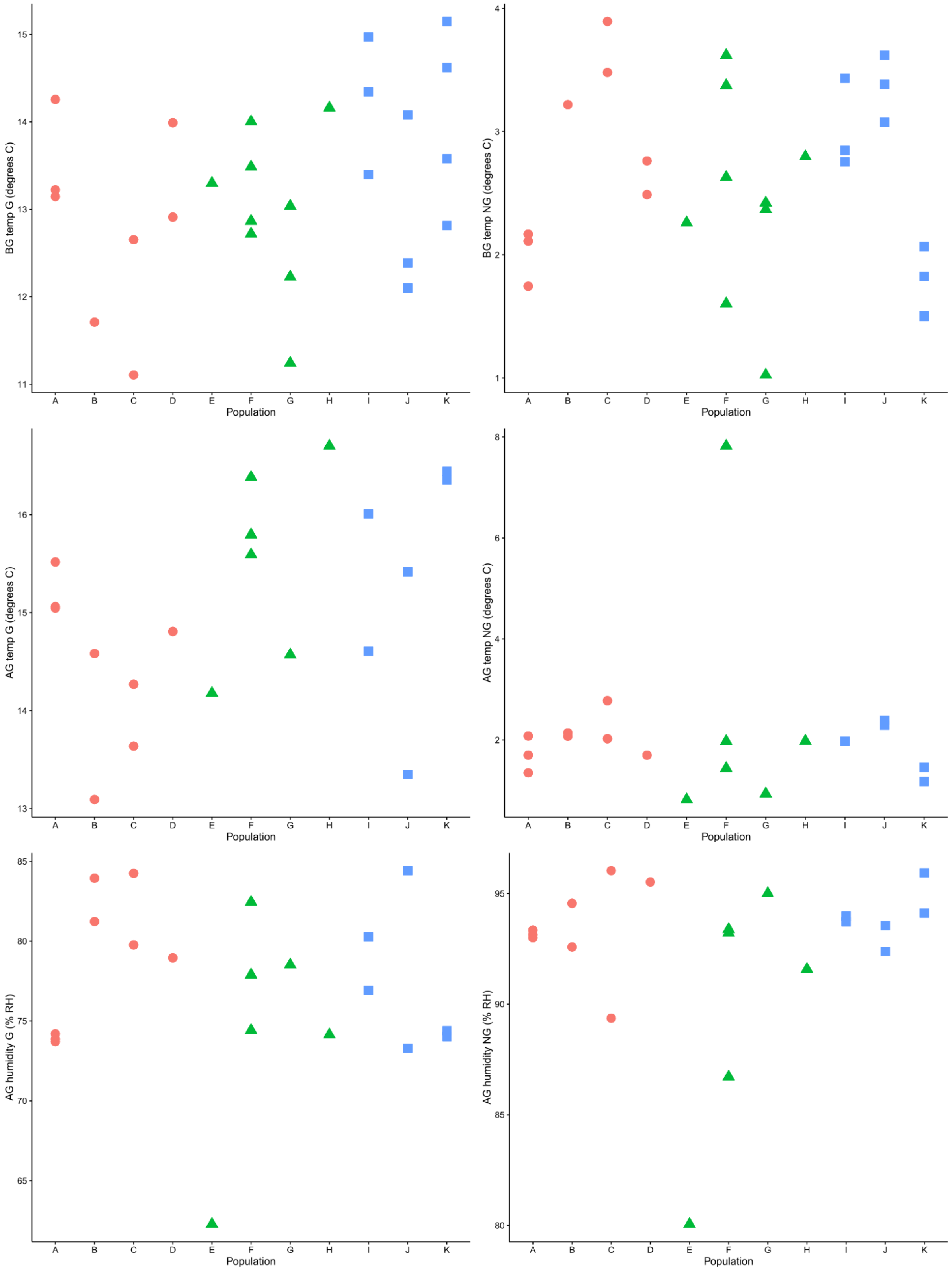

BG temp G (degrees C)
Population
BG temp NG (degrees C)
Population
AG temp G (degrees C)
Population
AG temp NG (degrees C)
Population
AG humidity G (% RH)
Population
AG humidity NG (% RH)
Population

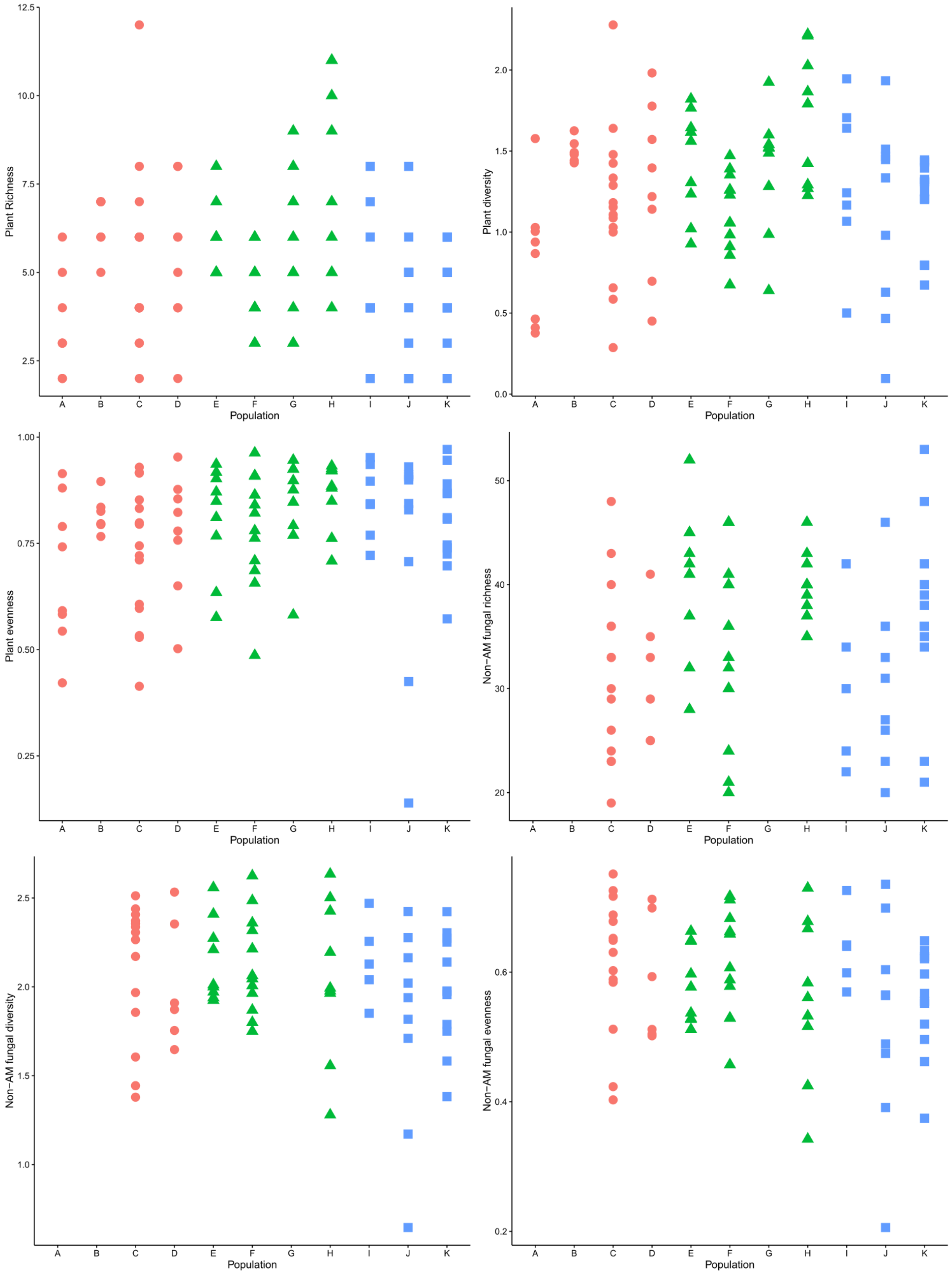
Plant Richness
Plant diversity
Plant evenness
Non−AM fungal richness
Non−AM fungal diversity
Non−AM fungal evenness
Population
A
B
C
D
E
F
G
H
I
J
K

Fig. S5 The relative importance of space and the environment in structuring the AM fungal community descriptors in the roots and root-associated soil. All values are presented using the adjusted coefficient of determination ($R^2_{adjusted}$). The variation is partitioned into four fractions: purely environmental variation, purely spatial variation, both environmental and spatial variation, and unexplained variation (residuals). All fractions that had an $R^2_{adjusted}$ smaller than 1% were not plotted. An asterisk indicates that a significant amount of variation is explained by the given fraction (P < 0.05).

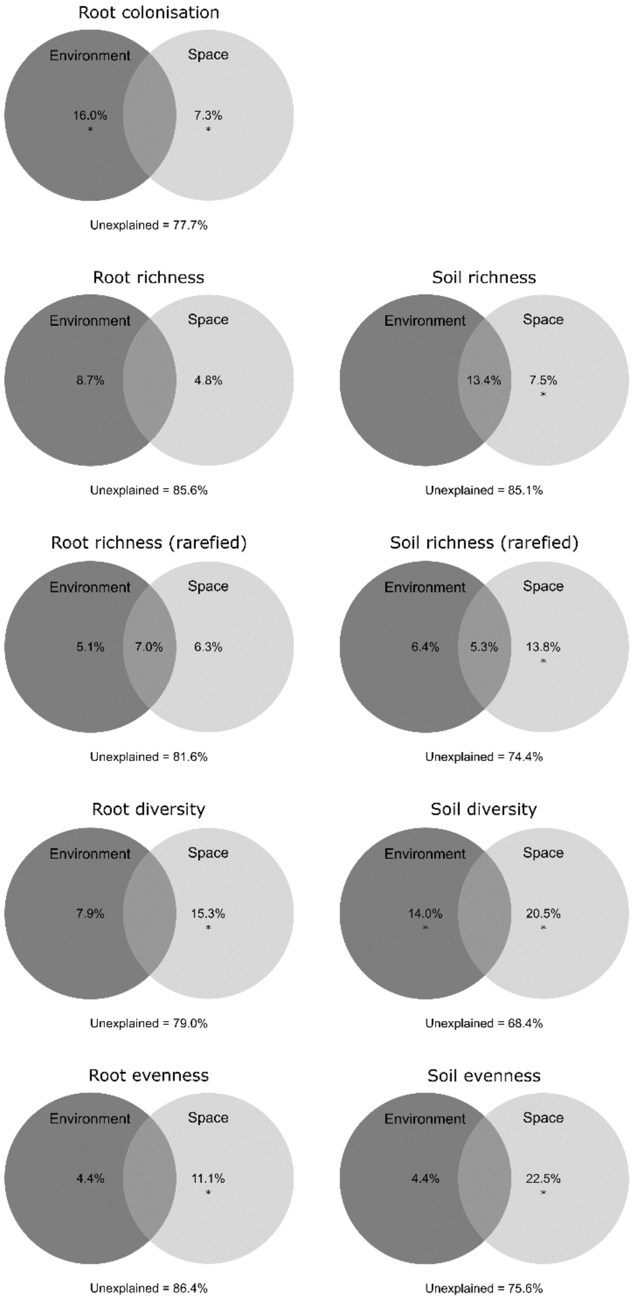

Fig. S6 Violin plot showing the distance between AM fungal communities in root and root-associated soil for each hierarchical spatial scale.

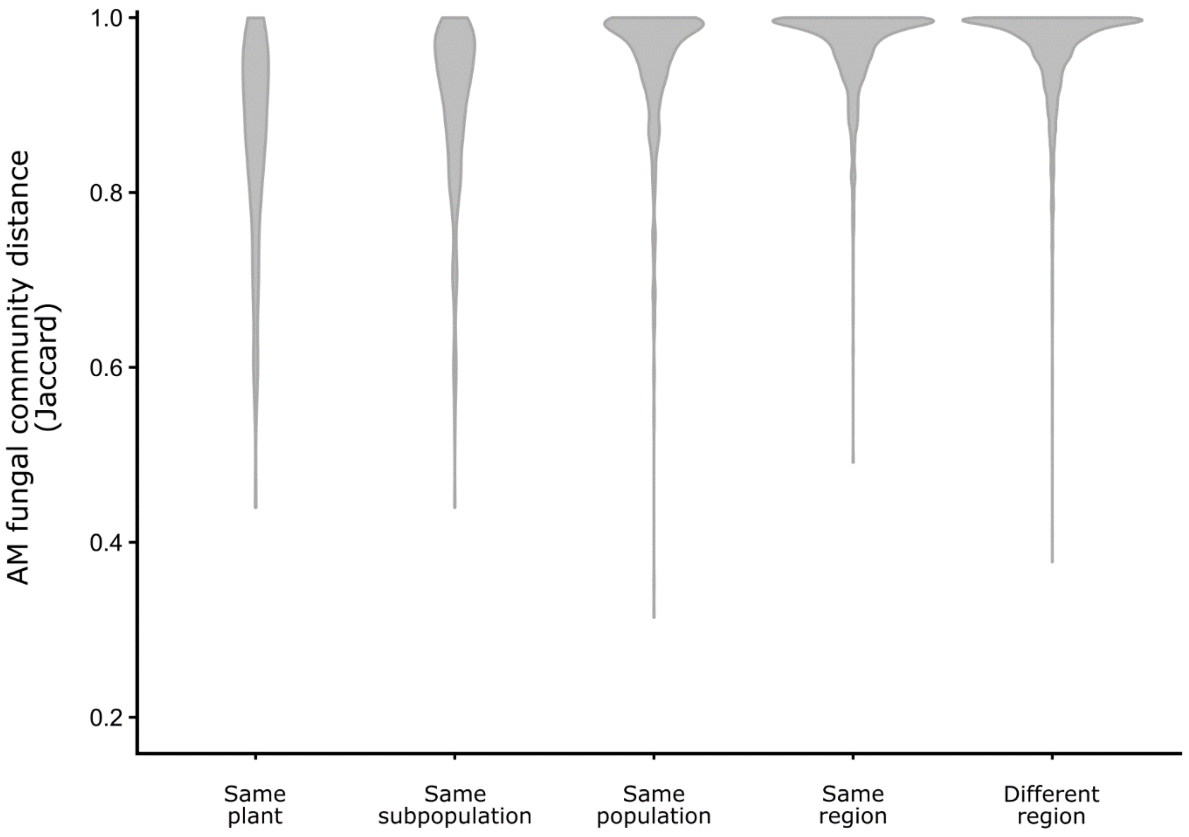

Table S1 ANOVA tables for the response variables AM fungal colonisation, AM fungal diversity indices, soil nutrients and bioclimatic variables. Models partitioned the variation in the response variables attributable to each of four (or three) hierarchical scales: regional, population, subpopulation and plant neighbourhood). Estimates of variation at the neighbourhood scale (AM fungal colonisation and diversity indices) and at the subpopulation scale (soil nutrients and bioclimatic variables) are based on the residuals from the models.

ANOVA	Sum of squares	DF	F	P	Variation explained (%)
**AM fungi**					
AM fungal colonisation					
Region	0.04009	2	0.7709	0.466394	1
Population	0.59457	8	2.858	0.008122	14.7
Subpopulation	1.52807	25	2.3504	0.002598	37.9
Plant neighbourhood (residuals)	1.87234	72			46.4
Residual richness (root)					
Region	8270	2	2.1359	0.126	3.9
Population	23838	8	1.5391	0.1602	11.2
Subpopulation	48188	25	0.9956	0.4848	22.7
Plant neighbourhood (residuals)	131648	68			62.1
Rarefied richness (root)					
Region	6114	2	3.6664	0.03075	6
Population	17325	8	2.5972	0.01539	17.1
Subpopulation	21385	25	1.0259	0.44902	21.1
Plant neighbourhood (residuals)	56700	68			55.8
Residual richness (soil)					
Region	75	2	0.0708	0.9317071	0.1
Population	17175	8	4.0738	0.0006037	28.8
Subpopulation	9675	23	0.7982	0.7204563	16.2
Plant neighbourhood (residuals)	32674	62			54.8
Rarefied richness (soil)					
Region	8000	2	5.7168	0.005265	7.9

Population	18664	8	3.3343	0.003101	18.4
Subpopulation	31641	23	1.9661	0.018466	31.1
Plant neighbourhood (residuals)	43383	62			42.7
<u>Residual diversity (root)</u>					
Region	0.4419	2	0.8123	0.4481091	1.2
Population	8.6878	8	3.9923	0.0006304	22.7
Subpopulation	10.5893	25	1.5572	0.077205	27.7
Plant neighbourhood (residuals)	18.497	68			48.4
<u>Residual diversity (soil)</u>					
Region	0.1308	2	0.3629	0.69711	0.5
Population	7.7318	8	5.3631	3.90E-05	29.4
Subpopulation	7.2365	23	1.7459	0.04288	27.5
Plant neighbourhood (residuals)	11.1729	62			42.5
<u>Residual evenness (root)</u>					
Region	0.01902	2	0.9993	0.373479	1.5
Population	0.24356	8	3.1986	0.003858	18.6
Subpopulation	0.39924	25	1.6777	0.04818	30.5
Plant neighbourhood (residuals)	0.64725	68			49.4
<u>Residual evenness (soil)</u>					
Region	0.00071	2	0.0351	0.9655404	0.1
Population	0.3951	8	4.9017	0.0001021	29.8
Subpopulation	0.30666	23	1.3233	0.1906515	23.1
Plant neighbourhood (residuals)	0.62469	62			47.1
**Abiotic factors**					
<u>pH</u>					
Region	0.00115	2	0.058	0.9437	0.1
Population	0.42228	8	5.3264	<0.001	35
Subpopulation (residuals)	0.78289	79			64.9
<u>P</u>					
Region	12.464	2	15.5434	<0.001	20.5

Population	16.711	8	5.2096	<0.001	27.5
Subpopulation (residuals)	31.676	79			52.1
<u>NH4</u>					
Region	15.211	2	12.707	<0.001	13.5
Population	49.769	8	10.394	<0.001	44.3
Subpopulation (residuals)	47.283	79			42.1
<u>NO3</u>					
Region	75.473	2	228.226	<0.001	48.4
Population	67.414	8	50.964	<0.001	43.2
Subpopulation (residuals)	13.062	79			8.4
<u>Total N</u>					
Region	24.345	2	137.869	<0.001	50.6
Population	16.833	8	23.832	<0.001	35
Subpopulation (residuals)	6.975	79			14.5
<u>Belowground temperature growing season</u>					
Region	12.334	2	11.9554	<0.001	14.3
Population	37.746	8	9.1467	<0.001	43.8
Subpopulation (residuals)	36.109	70			41.9
<u>Belowground temperature non-growing season</u>					
Region	0.9851	2	2.5021	0.08922	1
Population	31.2541	8	19.8455	<0.001	33
Subpopulation (residuals)	13.7801	70			14.5
<u>Aboveground temperature growing season</u>					
Region	13.217	2	21.98	<0.001	20.9
Population	35.214	8	14.64	<0.001	55.8
Subpopulation (residuals)	14.732	49			23.3
<u>Aboveground temperature non-growing season</u>					
Region	4.03	2	1.2828	0.28641	3.4
Population	35.864	8	2.854	0.01098	30.7
Subpopulation (residuals)	76.968	49			65.9

Aboveground relative humidity growing season					
Region	147.84	2	10.6	<0.001	9.5
Population	1064.06	8	19.073	<0.001	68.5
Subpopulation (residuals)	341.7	49			22
Aboveground relative humidity non-growing season					
Region	171.9	2	25.296	<0.001	22.7
Population	419.94	8	15.449	<0.001	55.4
Subpopulation (residuals)	166.49	49			22
Soil moisture					
Region	6845.5	2	36.5778	<0.001	33.1
Population	5335	8	7.1267	<0.001	25.8
Subpopulation (residuals)	8515.3	91			41.1
Plant richness					
Region	0.7953	2	4.1447	0.019782	3.8
Population	3.5675	8	4.6479	<0.001	17.2
Subpopulation	9.418	25	3.9265	<0.001	45.5
Plant neighbourhood (residuals)	6.9079	72			33.4
Plant diversity					
Region	0.8643	2	4.6157	0.0129987	4.2
Population	3.2562	8	4.3472	<0.001	15.9
Subpopulation	9.596	25	4.0996	<0.001	46.9
Plant neighbourhood (residuals)	6.7412	72			33
Plant evenness					
Region	0.10266	2	3.0512	0.05346	4.4
Population	0.1539	8	1.1435	0.34545	6.7
Subpopulation	0.84024	25	1.9977	0.01212	36.4
Plant neighbourhood (residuals)	1.21131	72			52.5
Non-AM fungal residual richness					
Region	126.15	2	1.8621	0.166175	3.5

Population	797.22	5	4.7072	0.001372	22.4
Subpopulation	976.64	19	1.5175	0.121095	27.4
Plant neighbourhood (residuals)	1659.75	49			46.6
Non-AM fungal residual diversity					
Region	0.5018	2	2.297	0.11129	5
Population	0.3889	5	0.7121	0.61728	3.9
Subpopulation	3.704	19	1.7848	0.05295	37.2
Plant neighbourhood (residuals)	5.352	49			53.8
Non-AM fungal evenness					
Region	0.029039	2	2.2753	0.113519	4.3
Population	0.042729	5	1.3392	0.263639	6.4
Subpopulation	0.284194	19	2.344	0.008619	42.5
Plant neighbourhood (residuals)	0.312681	49			46.8

Table S2 PERMANOVA table for the response variables AM fungal, plant, and non-AM root-associated fungal community composition. Models partitioned the variation in the response variables attributable to each of four hierarchical scales: regional, population, subpopulation and plant neighbourhood). Estimates of variation at the neighbourhood scale are based on the residuals from the models.

**PERMANOVA**	**DF**	**Sum of squares**	**Mean squares**	**F**	**R^2**	**P**	**Variation explained (%)**
**AM fungi**							
AM fungal community composition presence absence (root)							
Region	2	2.851	1.42534	2.5228	0.03906	0.001	3.9
Population	8	10.495	1.31191	2.322	0.14381	0.001	14.4
Subpopulation	25	20.884	0.83536	1.4786	0.28616	0.001	28.6
Plant neighbourhood (residuals)	67	37.854	0.56499	0.5187			51.9
Sample reads	1	0.896	0.89552	1.585	0.01227	0.002	1.2
AM fungal community composition presence absence (soil)							
Region	2	2.639	1.31972	1.9482	0.03399	0.001	3.4
Population	8	10.788	1.34845	1.9906	0.13893	0.001	13.9
Subpopulation	23	21.618	0.93992	1.3875	0.27841	0.001	27.8
Plant neighbourhood (residuals)	61	41.322	0.67741	0.53216			53.2
Sample reads	1	1.282	1.28243	1.8931	0.01652	0.001	1.7
AM fungal community composition abundance (root)							
Region	2	2.94	1.46984	3.6897	0.04347	0.001	4.3
Population	8	13.826	1.72831	4.3385	0.20445	0.001	20.4
Subpopulation	25	23.479	0.93916	2.3575	0.34719	0.001	34.7
Plant neighbourhood (residuals)	67	26.69	0.39836	0.39467			39.6
Sample reads	1	0.691	0.69087	1.7343	0.01022	0.013	1.0
AM fungal community composition abundance (soil)							
Region	2	3.254	1.62712	2.8971	0.04322	0.001	4.3

Population	8	13.751	1.71887	3.0604	0.18263	0.001	18.3
Subpopulation	23	23.212	1.00924	1.797	0.30829	0.001	30.8
Plant neighbourhood (residuals)	61	34.26	0.56164	0.45502			45.5
Sample reads	1	0.816	0.81583	1.4526	0.01084	0.077	1.1
**Biotic factors**							
Plant community composition							
Region	2	5.109	2.55429	5.6814	0.06214	0.001	6.2
Population	8	14.812	1.85155	4.1183	0.18016	0.001	18
Subpopulation	25	29.925	1.19699	2.6624	0.36398	0.001	36.4
Plant neighbourhood (residuals)	72	32.371	0.44959	0.39372			39.4
Non-AM fungal community composition							
Region	2	2.054	1.02688	2.2284	0.04306	0.001	4.3
Population	5	5.765	1.15306	2.5022	0.12089	0.001	12.1
Subpopulation	19	17.293	0.91014	1.9751	0.36259	0.001	36.3
Plant neighbourhood (residuals)	49	22.58	0.46082	0.47346			47.3

Table S3 Percentage of variation explained at each spatial scale for soil nutrients, bioclimatic variables, vegetation and non-AM root-associated fungi using either ANOVA (soil nutrients and bioclimatic variables) or PERMANOVA (vegetation and non-AM root-associated fungi).

	Soil nutrients					Temperature				Humidity			Vegetation				Non-AMF root-associated fungi			
	pH	P	NH_4	NO_3	Total N	BG (G)	BG (NG)	AG (G)	AG (NG)	Rel. (G)	Rel. (NG)	Soil moisture	Richness	Diversity	Evenness	Compo-sition	Richness	Diversity	Evenness	Compo-sition
Region	0.1	**20.5**	**13.5**	**48.4**	**50.6**	**14.3**	2.1	**20.9**	3.4	**9.5**	**22.7**	**33.1**	**3.8**	**4.2**	4.4	**6.2**	**8.6**	4.3	3.8.0	**4.3**
Population	**35.0**	**27.5**	**44.3**	**43.2**	**35.0**	**43.8**	**67.9**	**55.8**	**30.7**	**68.5**	**55.4**	**25.8**	**17.2**	**15.9**	6.7	**18.0**	**13.0**	4.7	7.4	**12.1**
Subpopulation*	64.9	52.1	42.1	8.4	14.5	41.9	29.9	23.3	65.9	22.0	22.0	41.1	**45.5**	**46.9**	**36.4**	**36.4**	**35.4**	37.4	**43.3**	**36.3**
Plant neighbourhood*	-	-	-	-	-	-	-	-	-	-	-	-	33.4	33.0	52.5	39.4	42.9	53.5	45.5	47.3

Significant estimates (P < 0.05) are shown in bold. G, growing season; NG, non-growing season.

* Estimates of variation at the subpopulation scale (soil nutrients and bioclimatic variables) and plant neighbourhood scale (vegetation and non-AM root-associated fungi) are based on the residuals from the models. Full model outputs can be found in Tables S1 and S2.

Table S4 P-values for the impact of environmental variables on the AM fungal root colonisation (%), richness, diversity and evenness in both roots and soil.

	Root colonisation	Richness		Diversity		Evenness	
		Root	Soil	Root	Soil	Root	Soil
pH	0.670	0.896	0.441	0.146	**0.043 (+)**	**0.020 (+)**	**0.038 (+)**
P	0.644	0.473	0.782	0.224	**0.038 (+)**	0.390	0.152
NH_4	0.276	0.730	0.728	0.933	0.155	0.720	0.176
NO_3	0.108	0.999	0.174	0.380	0.517	0.147	0.110
BG temperature (G)	0.994	0.547	0.500	0.150	0.093 (+)	0.271	0.302
BG temperature (NG)	0.876	0.911	0.189	**0.044 (-)**	0.442	**0.012 (-)**	0.559
AG temperature (G)	0.746	0.286	0.807	**0.029 (-)**	0.792	**0.014 (-)**	0.536
AG temperature (NG)	0.983	0.753	0.797	0.169	0.248	0.053 (+)	0.589
Annual relative humidity	0.505	0.419	0.359	0.491	0.605	0.153	0.332
Soil moisture	**0.006 (+)**	0.925	0.466	0.261	0.683	**0.017 (-)**	0.082 (-)
Plant richness	0.150	0.801	0.579	0.530	0.196	0.473	0.835
Root-associated fungal richness	0.760	0.921	0.673	0.872	0.959	0.628	0.159

Significant factors ($P < 0.05$) are given in bold, with the direction of the effect shown as (+) or minus (-). AG, aboveground; BG, belowground; G, growing season; NG, non-growing season.
